# Supplementary material for: Transport of antibody into the skin is only partially dependent upon the neonatal Fc-receptor
Source: PLoS One. 2023 Apr 24;18(4):e0273960. doi: 10.1371/journal.pone.0273960 (PMC10124839; doi:10.1371/journal.pone.0273960)
Supplement: S1 Fig — Wild-type and FcRn -/- mice were inoculated IV with 50 μg of anti-Langerin mAb. Twenty-four hours later, mouse ears were excised, fixed, and stained with a secondary antibody and ears were imaged by confocal microscopy. (A) Maximum projection images of stained Langerhans cells from control and FcRn -/- mice. (B) Total number of stained Langerhans cells in control and FcRn -/- mouse ears counted using the spot function in Imaris. In contrast to the data shown in Figs 1 and 2, which shows fluorescence intensity, these data show total number of spot counts and indicate that there is no difference in the total number of Langerhans cells between Wild-type and FcRn -/- mice. Statistical comparison was performed using an unpaired t-test (two-tailed, α = 0.05). (PDF) [file pone.0273960.s001.pdf]

Supporting Figure 1

A

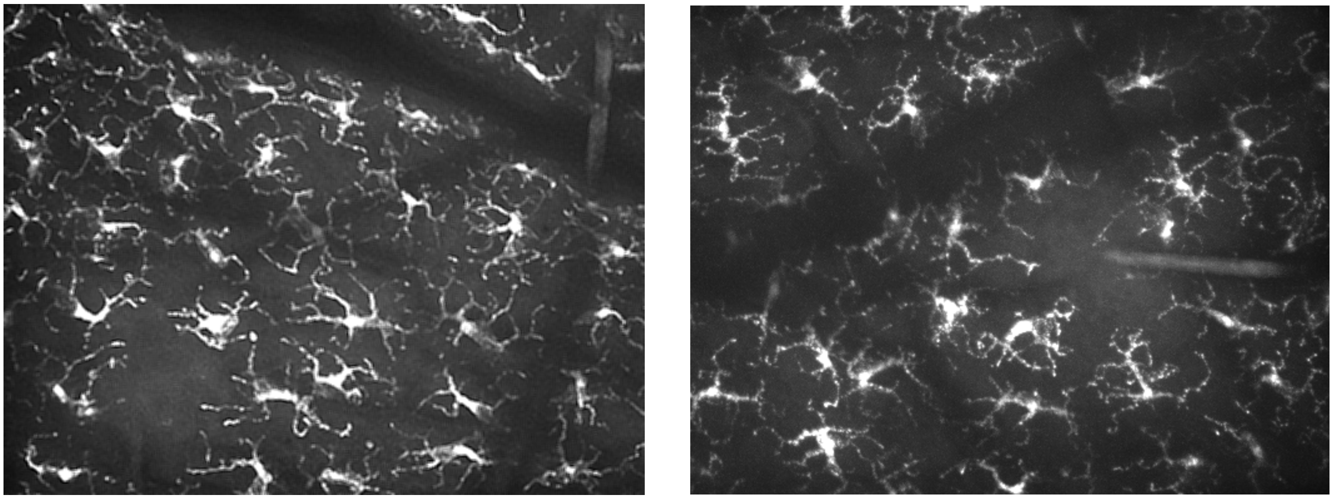

B

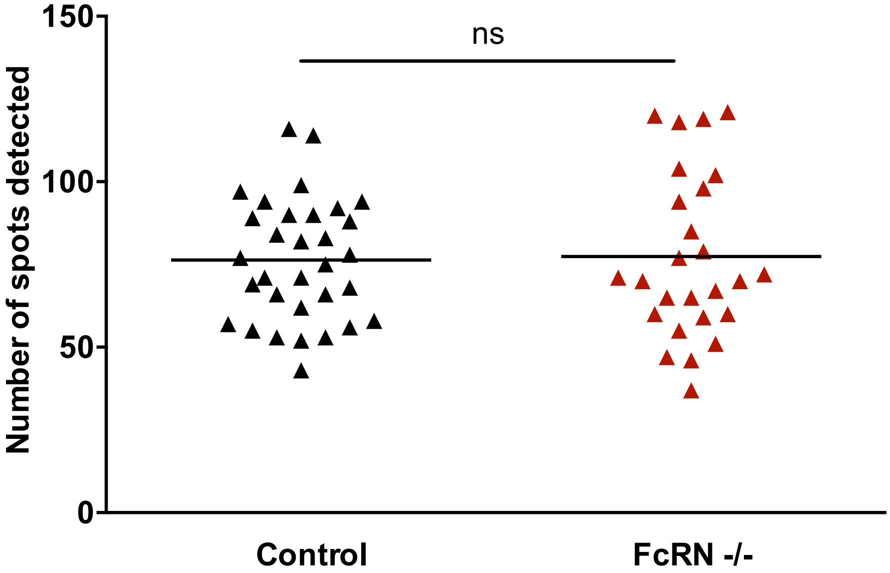

Figure 1. Staining of Langerhans cells in FcRn -/- and Control mice. Wild-type and FcRn -/- mice were inoculated IV with 50 µg of anti-Langerin mAb. Twenty-four hours later, mouse ears were excised, fixed, and stained with a secondary antibody and ears were imaged by confocal microscopy. (A) Maximum projection images of stained Langerhans cells from control and FcRn -/- mice. (B) Total number of stained Langerhans cells in control and FcRn -/- mouse ears counted using the spot function in Imaris. In contrast to the data shown in Figures 1 and 2, which shows fluorescence intensity, these data show total number of spot counts and indicate that there is no difference in the total number of Langerhans cells between Control and FcRn -/- mice. Statistical comparison was performed using an unpaired t-test (two-tailed, α=0.05).
