# Supplementary material for: Transport of antibody into the skin is only partially dependent upon the neonatal Fc-receptor
Source: PLoS One. 2023 Apr 24;18(4):e0273960. doi: 10.1371/journal.pone.0273960 (PMC10124839; doi:10.1371/journal.pone.0273960)
Supplement: S2 Fig — (A) P. berghei sporozoite infectivity in C57Bl/6J and C57Bl/6N mice after intravenous or mosquito bite inoculation of parasites. Mice were intravenously inoculated with 350 P. berghei sporozoites or exposed to 8 infected mosquito bites and 40 hours later, livers were harvested and the level of Pb 18s rRNA in the liver quantified by RT-qPCR. Shown are the pooled data from two independent experiments (n = 4–5 mice per experiment). Statistical comparisons between C57Bl/6J and C57Bl/6N mice were performed on the pooled data using two-tailed Mann-Whitney tests. The difference between the groups was not statistically significant (ns, p>0.05). (B) P. berghei sporozoite infectivity in C57Bl/6J wild-type and FcRn -/- mice after intravenous and mosquito bite inoculation of parasites. Mice were intravenously inoculated with 350 P.berghei sporozoites or exposed to 7 infected mosquito bites. Forty hours later, livers were harvested and the level of Pb 18s rRNA in the liver was quantified using RT-qPCR. Shown is data from one experiment (n = 3–4 mice per group). Statistical comparisons between wild-type and FcRn -/- mice were performed using two-tailed Mann-Whitney tests and were not significant (ns, p>0.05). (PDF) [file pone.0273960.s002.pdf]

Supporting Figure 2

A

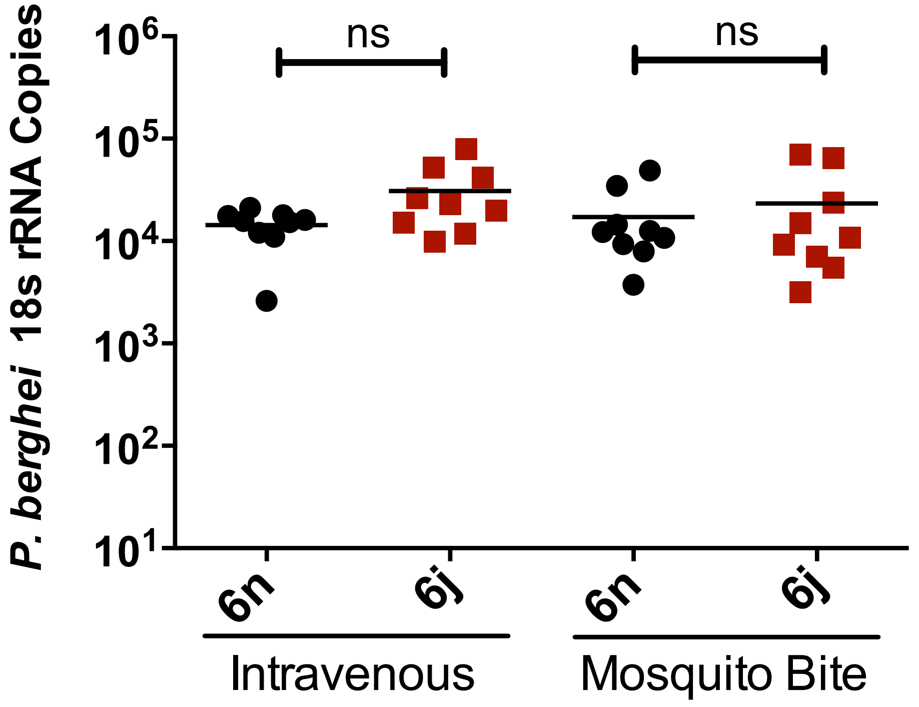

B

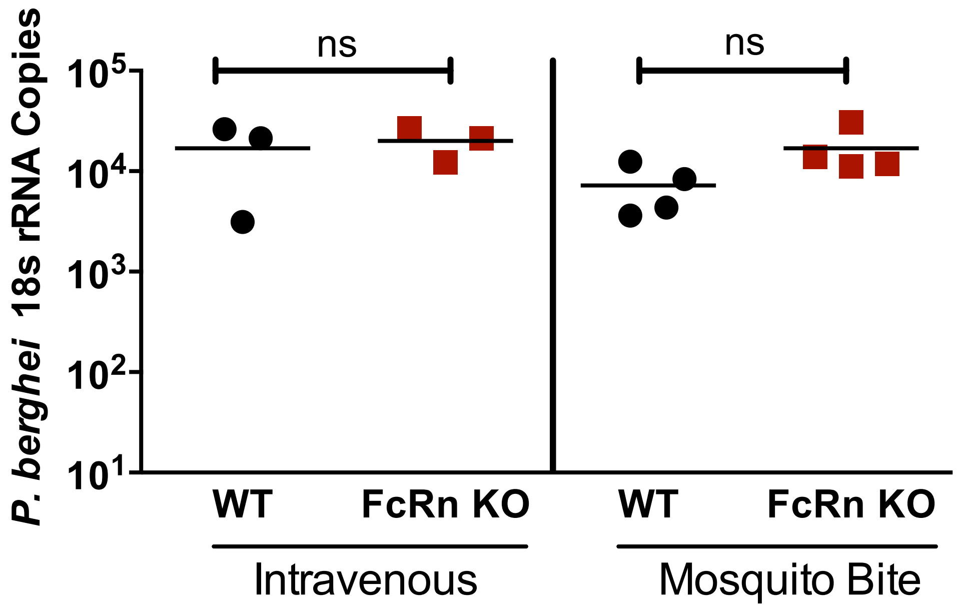

Supplemental Figure 2. Sporozoite infectivity in C57Bl/6J and C57Bl/6N mice and C57Bl/6J wild-type and FcRn -/- mice. (A). *P. berghei* sporozoite infectivity in C57Bl/6J and C57Bl/6N mice after intravenous or mosquito bite inoculation of parasites. Mice were intravenously inoculated with 350 *P. berghei* sporozoites or exposed to 8 infected mosquito bites and 40 hours later, livers were harvested and the level of Pb 18s rRNA in the liver quantified by RT-qPCR. Shown are the pooled data from two independent experiments (n=4-5 mice per experiment). Statistical comparisons between C57Bl/6J and C57Bl/6N mice were performed on the pooled data using two-tailed Mann-Whitney tests. The difference between the groups was not statistically significant (ns, $p>0.05$). **(B)**. *P. berghei* sporozoite infectivity in C57Bl/6J wild-type and FcRn -/- mice after intravenous and mosquito bite inoculation of parasites. Mice were intravenously inoculated with 350 *P.berghei* sporozoites or exposed to 7 infected mosquito bites. Forty hours later, livers were harvested and the level of Pb 18s rRNA in the liver was quantified using RT-qPCR. Shown is data from one experiment (n=3-4 mice per group). Statistical comparisons between wild-type and FcRn -/- mice were performed using two-tailed Mann-Whitney tests and were not significant (ns, $p>0.05$).
