# Supplementary material for: Transport of antibody into the skin is only partially dependent upon the neonatal Fc-receptor
Source: PLoS One. 2023 Apr 24;18(4):e0273960. doi: 10.1371/journal.pone.0273960 (PMC10124839; doi:10.1371/journal.pone.0273960)
Supplement: Attachment — Submitted filename: A_ResponseToReviewers_Final.docx [file pone.0273960.s003.docx]

We thank the Reviewers and Editor for their helpful comments on our manuscript. They raised good points, which we have addressed as outlined below.

Editor Comment:

I also noted that one sentence in the abstract is misleading and should be revised ("the protective efficacy of a passively-administered antibody is reduced in the skin in FcRn deficient mice, but not to the same extent that we have previously observed..." (Protection is reduced as compared to WT).

We’re not exactly sure what needed to be changed but think it's the reference to antibody in the skin. We have changed this sentence as follows below but if we misinterpreted please let us know and we will change accordingly.

*To understand the relevance of FcRn in the context of malaria infection, we use the rodent parasite Plasmodium berghei and show that the protective efficacy of a passively-administered anti-malarial antibody is reduced in FcRn deficient mice, but not to the same extent as previously observed in wildtype mice.*

Reviewer #1:

There are two points regarding figure 3 that still need explanation before the acceptation of the manuscript.

The first is that in figure 3, values are determined as a percentage of the amount of 3D11 mAb in the serum at 6h. It is not clear if all points are normalized to the value of WT group at 6h or by the respective 6h values. In the last case, despite the same normalized kinetics until 24h, it is not possible to appreciate the decrease in serum concentration that could help to explain the differences observed in figures 1, 2 and 4. Please plot the non-normalized data.

The values are normalized to the respective 6 hr values and this is now made clear in both the Figure 3 legend and the Results section:

Fig 3 legend: *The percentage of mAb 3D11 remaining at each time point in WT and FcRn -/- mice was determined as a ratio with the amount of serum mAb 3D11 at the respective 6 hr value.*

Results: *As shown in Figure 3A, when serum antibody concentration is normalized to the respective value in the first sample (at 6 hrs)…*

Thus, the reviewer is correct that any differences in clearance kinetics at early time points between in WT vs FcRn KO mice prior to 24 hrs might not be evident. We plotted the data this way because initial sampling can give variable results dependent upon mouse size and other factors, and we found that serum clearance studies are frequently reported this way (References 1-4 below). However, we agree with the reviewer that its good to show the non-normalized data which indicate, as the reviewer predicted, that the decreased serum concentration in the FcRn mice at the early time points could explain the differences observed in Figures 1, 2, and 4. We now include both normalized and non-normalized data in the revised Figure 3. We have also changed the text in several places to reflect this: Results section (page 5), legend for Figure 3, and Discussion (bottom of page 8).

Fig 3 legend: *(B) Non-normalized serum clearance data for WT and FcRn-/- mice is shown. Absorbance was measured in serial dilutions of serum and the dilution corresponding to an absorbance of 1.0 is shown. No comparisons between WT and FcRn-/- mice reached statistical significance.*

Results: *However, when non-normalized data are plotted the initial antibody levels in FcRn-/- mice are lower compared to WT controls (Fig 3B). This could be because the FcRn-/- mice were slightly older and possibly larger, or it could be due to increased antibody degradation due to decreased antibody recycling in the FcRn-/- mice. Indeed though the majority of studies on the recycling function of FcRn focus on the degradation of antibody over days, a few studies that included clearance kinetics over the first 24 hrs demonstrate that in FcRn-/- mice clearance of IgG from the circulation is increased in this time frame [26, 27]. In the first 24 hrs post inoculation, serum antibody levels in WT mice decrease rapidly likely because of equilibration with the extravascular compartment, possibly via macropinocytosis, which is likely also occurring in the FcRn-/- mice. In a previous study comparing sporozoite infectivity in passively immunized WT mice challenged by either mosquito bite or IV inoculation, we allowed for antibody equilibration prior to challenge. Thus to compare our current results to that study, we selected 22 hrs after antibody inoculation as the timepoint to challenge the FcRn-/- mice.*

Discussion: *Overall, our data demonstrate that FcRn may have some role in antibody transport into the skin, though other transport mechanism(s) also function in this process. This is suggested by two observations: 1) the decreased amount of anti-langerin IgG in the skin of FcRn-/- mice, as is evidenced by the decreased staining of Langerhans cells, and 2) the decreased amount of mAb 3D11 in the skin, which is suggested by the decreased inhibitory activity on sporozoites observed in FcRn-/- mice. These findings could be due to increased destruction of antibody due to the lack of FcRn or to the decreased transfer of antibody into the skin via FcRn, or both. In either case, given our current findings and previous studies demonstrating an important role of dermal antibody in protection against malaria sporozoites [8,9] , it is possible that increasing the half-life of these antibodies could improve current vaccine efforts targeting sporozoites.*

The second is that a quick search about mAb clearance curve in FcRn-/- mice showed that at a substantial decrease in the amount of antibodies can be observed at 24h (PMID: 27610650, PMID: 28613103). Please compare/discuss your data with published data.

We actually did not mean to imply that antibody levels do not decrease in the first 24 hrs. In fact in both WT and FcRn-/- mice this is a time of greatest decrease in serum levels largely because of antibody equilibration with the extravascular spaces. However, in line with what the reviewer is saying, when we plot the non-normalized data, its clear that there is less serum antibody in FcRn-/- mice even at early time points. We now have added the following sentence to the Results section (page 5) and cite additional references *(References 5 and 6 below and in paper new References 26 and 27)* to reflect this:

*Indeed though the majority of studies focused on the recycling function of FcRN focus on the degradation of antibody over days, a few studies that included clearance kinetics over the first 24 hrs demonstrate that in FcRN-/- mice clearance of IgG from the circulation is increased in FcRN-/- in this time* *frame [26, 27].*

Reviewer #2: It would be good to address why the authors did not use FcRn binding site knockouts to test this from the antibody side. That would likely be a cleaner way to address this, by knocking out FcRN binding of the antibody, and may have fewer pleiotropic effects than the genetic mouse knockout. Perhaps a discussion of this would suffice, but it is an obvious question an erudite reader will ask.

One can digest antibodies with papain and obtain Fab fragments that do not contain the Fc portion. Though many experiments have been done with these Fab fragments, when they are inoculated into the blood circulation, they are rapidly cleared (Reference 7 below). Indeed in the case of mAb 3D11 that we used in this paper, a previous study testing Fab fragments had to preincubate sporozoites with the Fabs in vitro prior to IV inoculation specifically because they were cleared so quickly once in the blood that they could not be tested by passively immunizing the mice (Reference 8 below). Nonetheless, there are now many studies in which, the Fc regions are changed to investigate binding to the FcRN receptor. We have now included additional references citing these studies in the Discussion (Reference 9 & 10 below which are added as References 33 and 34 in the paper)**:**

*Through a rational design of IgG-based therapeutics, the half-life of molecules such as antibodies and Fc-drug conjugates can be prolonged in the serum by several fold, and the amount deposited into tissues significantly enhanced (33, 34, 35 and reviewed in [36]).*

Minor points:
All log scale graphs lack minor ticks

Log scale graphs now have minor ticks

bottom of 2C was cutoff in the review copy

We have tried to correct this

Fluorescence units were not listed or labeled, or defined in the M/M

We have now corrected this in the Methods and Figure legends:

Methods: … *and the sum fluorescence intensity measured using relative fluorescence units (RFU) for each spot quantified. This is the fluorescence signal detected by the instrument. Identical exposure times were used for all images.*

Fig 1 and 2 Legends: *The sum fluorescence was calculated using relative fluorescence units (RFU), with each spot representing the mean sum fluorescence in one image. Identical exposure times were used for all images.*

References

1) Montoyo HP, Vaccaro C, Hafner M, Ober RJ, Mueller W, Ward ES. Conditional deletion of the MHC class I-related receptor FcRn reveals the sites of IgG homeostasis in mice. Proc Natl Acad Sci U S A. 2009 Feb 24;106(8):2788-93. doi: 10.1073/pnas.0810796106. PMID: 19188594.
2) Roopenian DC, Christianson GJ, Sproule TJ, Brown AC, Akilesh S, Jung N, Petkova S, Avanessian L, Choi EY, Shaffer DJ, Eden PA, Anderson CL. The MHC class I-like IgG receptor controls perinatal IgG transport, IgG homeostasis, and fate of IgG-Fc-coupled drugs. J Immunol. 2003 Apr 1;170(7):3528-33. doi: 10.4049/jimmunol.170.7.3528. PMID: 12646614.
3) Junghans RP, Anderson CL. The protection receptor for IgG catabolism is the beta2-microglobulin-containing neonatal intestinal transport receptor. Proc Natl Acad Sci U S A. 1996 May 28;93(11):5512-6. doi: 10.1073/pnas.93.11.5512. PMID: 8643606
4) Israel EJ, Wilsker DF, Hayes KC, Schoenfeld D, Simister NE. Increased clearance of IgG in mice that lack beta 2-microglobulin: possible protective role of FcRn. Immunology. 1996 Dec;89(4):573-8. doi: 10.1046/j.1365-2567.1996.d01-775.x. PMID: 9014824
5) Montoyo HP, Vaccaro C, Hafner M, Ober RJ, Mueller W, Ward ES. Conditional deletion of the MHC class I-related receptor FcRn reveals the sites of IgG homeostasis in mice. Proc Natl Acad Sci U S A. 2009 Feb 24;106(8):2788-93. doi: 10.1073/pnas.0810796106. PMID: 19188594.
6) Eigenmann MJ, Fronton L, Grimm HP, Otteneder MB, Krippendorff BF. Quantification of IgG monoclonal antibody clearance in tissues. MAbs. 2017 Aug/Sep;9(6):1007-1015. doi: 10.1080/19420862.2017.1337619. PMID: 28613103
7) SPIEGELBERG HL, WEIGLE WO. THE CATABOLISM OF HOMOLOGOUS AND HETEROLOGOUS 7S GAMMA GLOBULIN FRAGMENTS. J Exp Med. 1965 Mar 1;121(3):323-38. doi: 10.1084/jem.121.3.323. PMID: 14271318.
8) Potocnjak P, Yoshida N, Nussenzweig RS, Nussenzweig V. Monovalent fragments (Fab) of monoclonal antibodies to a sporozoite surface antigen (Pb44) protect mice against malarial infection. J Exp Med. 1980 Jun 1;151(6):1504-13. doi: 10.1084/jem.151.6.1504. PMID: 6991628.
9) Mackness BC, Jaworski JA, Boudanova E, Park A, Valente D, Mauriac C, Pasquier O, Schmidt T, Kabiri M, Kandira A, Radošević K, Qiu H. Antibody Fc engineering for enhanced neonatal Fc receptor binding and prolonged circulation half-life. MAbs. 2019 Oct;11(7):1276-1288. doi: 10.1080/19420862.2019.1633883. PMID: 31216930
10) Ko S, Park S, Sohn MH, Jo M, Ko BJ, Na JH, Yoo H, Jeong AL, Ha K, Woo JR, Lim C, Shin JH, Lee D, Choi SY, Jung ST. An Fc variant with two mutations confers prolonged serum half-life and enhanced effector functions on IgG antibodies. Exp Mol Med. 2022 Nov;54(11):1850-1861. doi: 10.1038/s12276-022-00870-5. Epub 2022 Nov 1. PMID: 36319752.
